# Park renovations in low-income New York City neighborhoods and changes in adult physical activity and sedentary behavior: a quasi-experimental study

**DOI:** 10.1186/s12966-026-01958-1

**Published:** 2026-08-03

**Authors:** Rachel L. Thompson, Kelly R. Evenson, Katarzyna E. Wyka, Lorna E. Thorpe, Glen D. Johnson, Brian T. Pavilonis, Terry T.-K. Huang

**Affiliations:** 1 Center for Systems and Community Design, Graduate School of Public Health and Health Policy, City University of New York (CUNY), 55 West 125th Street, New York, NY 10027 USA; 2 NYU-CUNY Prevention Research Center, New York, NY USA; 3 Department of Environmental, Occupational, and Geospatial Health Sciences, Graduate School of Public Health and Health Policy, City University of New York (CUNY), 55 West 125th Street, New York, NY 10027 USA; 4 Department of Epidemiology, Gillings School of Global Public Health, University of North Carolina–Chapel Hill, 135 Dauer Drive, Chapel Hill, NC 27599 USA; 5 Department of Population Health, NYU Grossman School of Medicine, 180 Madison Ave, New York, NY 10016 USA

**Keywords:** Built environment, Urban health, Health equity, Parks, Park renovations, Quasi-experiment, Accelerometry, Physical activity, Sedentary behavior

## Abstract

**Background:**

Inadequate physical activity (PA) elevates chronic disease risk in low-income urban populations. Neighborhood parks may offer venues for PA, but effects likely depend on park quality and use patterns. This study examined whether citywide park renovations were associated with changes in adult PA and sedentary behavior (SB) in low-income New York City neighborhoods and whether associations between park use and PA/SB changes differed for residents near renovated vs. control parks.

**Methods:**

We conducted a quasi‑experimental prospective cohort study (2016–2022) in neighborhoods surrounding 31 renovated and 21 matched control parks. Participants included 344 adults living ≤ 0.3 miles of a study park (186 intervention, 158 control). Outcomes were accelerometry‑based PA/SB measures collected pre‑ and post‑renovation: vector magnitude per minute (VMPM) and minutes/day of sedentary time, light PA, and moderate‑to‑vigorous PA. Surveys assessed self‑reported typical frequency and duration of park visits. Linear mixed‑effects difference-in-differences models estimated associations between renovations and PA/SB changes; interaction models examined associations of park use with PA/SB changes in intervention vs. control groups.

**Results:**

Changes in PA/SB were not significantly different between intervention and control groups. However, intervention participants who reported visiting their study park ≥ 30 min on a weekend day experienced a 140.0-unit/day greater increase in VMPM (95% CI 52.7,227.2) and a 61.1-minute/day larger decrease in sedentary time (95% CI -108.8,-13.3) compared to those with shorter visit durations. Furthermore, in the intervention group, each additional 60 min spent at a study park on weekdays was associated with 18.0 more minutes/day of light PA (95% CI 4.7,31.3), and each additional 60 min spent at the study park on weekend days corresponded to 19.0 more minutes/day of light PA (95% CI 8.1,29.9) and a 54.1-unit increase in VMPM/day (95% CI 7.5,100.7). No associations between park use duration and PA/SB changes were observed in the control group.

**Conclusions:**

Park renovations alone were not associated with PA/SB changes, but longer duration of visits at renovated—but not control—parks were associated with increased total PA volume and light PA, and decreased sedentary time. High-quality park environments may be a necessary pre-condition to the benefits of park use on adult PA/SB.

**Supplementary Information:**

The online version contains supplementary material available at https://doi.org/10.1186/s12966-026-01958-1.

## Background

Inadequate physical activity (PA) is a significant contributor to global morbidity and mortality, accounting for an estimated 7.2% of all-cause deaths and 7.6% of cardiovascular disease deaths in 2016 [1]. Longitudinal studies consistently show that infrequent PA and frequent sedentary behavior (SB) increase risk of non-communicable diseases, including obesity, cardiovascular disease, diabetes, depression, and dementia [2–4]. Between 2017 and 2020 in the United States (US), 25.3% of adults reported no PA outside of their regular job in the past month [5]. Moreover, in 2019 US adults reported an average of 9.5 h/day of sedentary time, corresponding to nearly 60% of typical waking hours [6]. National data reveal persistent disparities in PA/SB across sociodemographic groups, with racial/ethnic minorities and low-income adults exhibiting the highest rates of inadequate PA [5, 7].

Urban parks are increasingly recognized as important built environment features that support PA across diverse demographic groups [8–11]. Parks offer free, accessible venues for leisure-time PA, particularly in low-income neighborhoods where private recreational opportunities are limited [12, 13]. Amenities such as walking paths, sports courts, and fitness equipment may facilitate PA behaviors [14, 15]. However, the extent to which urban parks support PA is likely contingent upon their quality, as multiple studies have shown that parks in disrepair or with incivilities are less utilized and may be negatively associated with health and quality of life [16–20]. Disparities in access to high-quality parks persist across urban settings, with low-income neighborhoods having fewer parks that are often underutilized and poorly maintained compared to those in higher-income neighborhoods [12, 21, 22].

Park renovations may be one means of increasing PA and reducing SB among low-income urban populations. Numerous place-based intervention studies have shown positive associations between park improvements on the observed PA levels of park users [23]. However, the majority of past intervention studies have relied on systematic observations or intercept surveys of park users and fail to capture the effects of park improvements on individual-level changes over time using accelerometry-based measures of PA/SB [10, 23]. Only a few studies to date have evaluated changes in the PA levels of residents living near parks receiving improvements, all of which showed either null or mixed findings [24–26]. As a result, there is a significant gap in our understanding of how park improvements may influence PA and SB of individuals living in the neighborhoods surrounding parks.

In addition to park renovations, park use patterns represent a key behavioral mechanism through which environmental interventions may influence PA/SB outcomes. Growing evidence suggests that the dosage of park use matters, with studies showing better health outcomes are associated with greater frequency and/or duration of park use [27–30]. For example, in a recent study conducted among a population-representative sample of *n* = 2,000 residents of low-income neighborhoods in New York City (NYC), visiting a recently renovated park once per week or more was associated with a 30% higher prevalence of high self-reported PA levels compared to those who visited the park less than once per week [30]. Regarding duration of visits, a study of *n* = 1,538 Brisbane City, Australia residents found that visits to greenspaces of ≥ 30 min during the course of a week could reduce the population prevalence of depression by 7% and high blood pressure by 9% [28]. Similarly, another study among *n* = 19,806 United Kingdom residents found that ≥ 120 min of nature contact in the past week was associated with 59% higher likelihood of reporting good health and 23% higher likelihood of reporting high well-being compared to no nature contact [29].

The Community Parks Initiative (CPI) is an ongoing citywide effort in NYC to improve access to high-quality park spaces in low-income urban communities through park redesign and renovation [31]. Since 2014, nearly 70 parks have been renovated through the CPI [31], with many renovations completed between 2017 and 2021, which enabled a quasi-experimental evaluation of the CPI’s impact on changes in behaviors and health outcomes among residents of the neighborhoods surrounding the parks [32]. We previously found that CPI renovations were associated with positive changes in park usership [33], as well as the amount of time neighborhood residents typically spent at parks [34]. In this study, we assessed whether CPI renovations were associated with changes in accelerometry-measured PA/SB among adults living near renovated parks compared to matched control sites, and whether associations between park use patterns and PA/SB changes were stronger among those living near renovated parks vs. control parks.

## Methods

### Study design and setting

This study employed a quasi-experimental prospective cohort design to evaluate the effect of citywide park renovations on changes in adult PA and SB [32]. The analysis included participants from low-income NYC neighborhoods surrounding 31 intervention parks and 21 socio-demographically matched control parks. The study was conducted between 2016 and 2022. Eligible park sites were in neighborhoods meeting at least two of the following criteria: ≥ 20% of residents below federal poverty line, ≥ 25% population increase from 2000 to 2010, and ≥ 110 residents/acre. Sites must not have received more than US $250,000 in capital investment in the prior two decades. Matching of intervention and control sites was done using best frequency matches (± 6% difference) across aggregate neighborhood-level sociodemographic characteristics (percent of the population ≥ 18y, percent White residents, percent Black residents, percent Asian residents, percent Latino/a residents, percent change in population between 2000 and 2010, and percent of residents living below the federal poverty line) [32]. Control and intervention parks were not matched on specific baseline park characteristics. However, the study parks were generally small neighborhood parks, and park sizes were similar between the 31 intervention [median = 1.00 acres (IQR: 0.69–1.30 acres)] and 21 control parks [median = 1.00 acres (IQR: 0.65–1.25 acres)].

### Intervention

The CPI intervention, implemented by NYC Parks, involved extensive redesign and renovation of intervention parks, with a median capital investment of approximately US $4 million per park site for the intervention parks included in this study (IQR: $3 million - $5 million) [35]. Renovations at intervention parks were tailored to community inputs and site conditions [36], with common enhancements including expanded greenery, upgraded play and fitness equipment, improved walkways and accessibility features, updated sports courts and multipurpose fields, and aesthetic enhancements such as landscaping, shading, and seating [33]. Control parks met the same eligibility criteria as intervention parks, however they did not receive any redesign or renovation enhancements during the study period.

### Participants

Eligible participants were adult residents (≥ 18 years) living within a 0.3-mile radius of an intervention or control park, with no mobility limitations and fluency in English, Spanish, or Chinese. The study catchment radius was purposefully defined based on data collected by NYC Parks prior to study initiation, which indicated that 70% of park users traveled ≤ 5 city blocks (approximately ≤ 0.3 miles) to visit their neighborhood park [37]. This distance is also consistent with commonly used definitions of comfortably walkable distance in built environment and public health research in the US (i.e., a 5–10 min walk) [38, 39]. Recruitment methods for the study have been previously described in detail [32, 40, 41]. Briefly, multifaceted recruitment strategies were employed, including through recruitment events and street-intercept recruitment [40]. Outreach was led by trained study ambassadors in collaboration with NYC Parks’ *Partnership for Parks*, targeting both public housing and non-public housing residents [32, 40]. In addition, a variety of methods were employed to maximize retention of participants over time, including using multiple methods of contact, repeated follow-up attempts, and $50-$75 incentives for completion of study activities at each wave of data collection [40].

### Data collection

Data were collected in two waves (pre- and post-renovation) using surveys and accelerometry-based PA/SB monitoring. Surveys captured self-reported park use behaviors and sociodemographic characteristics, and were administered either digitally or on paper. PA/SB was measured using ActiGraph GT3X-BT accelerometer devices worn on the right hip during all waking hours except while bathing or swimming. Devices were worn for seven consecutive days pre- and post-renovation. Wear time and placement protocols followed established methods [42]. Baseline data were collected for both intervention and control groups between 2016 and 2018, and follow-up between 2018 and 2021. Data collection was conducted primarily between April and December for both pre- and post-renovation waves in both intervention and control groups (a timeframe generally conducive to outdoor activity and park use in NYC). Median follow-up time was 25 months post-baseline (IQR: 24–27 months) and was similar between intervention and control groups [intervention group median = 25 months (IQR: 24–28 months); control group median = 24 months (IQR: 23–26 months)]. In the intervention group, follow-up occurred six months (on average) after the renovated parks reopened.

### Measures

#### Physical activity outcomes

Accelerometry data were processed using ActiLife^®^ software by Ametris (https://ametris.com/actilife). Devices recorded activity counts in 15-second epochs. Non-wear time was defined as ≥ 90 consecutive minutes of zero counts, allowing up to two minutes of non-zero counts if no counts were detected during the 30 min upstream/downstream of that interval [43, 44]. Any non-zero counts (except allowed intervals) were considered wear time; counts during non-wear periods were set to missing.

Participants with ≥ 3 adherent days (≥ 8 h/day of wear time) both pre- and post-renovation were included. Outcome measures were averaged across adherent days at each wave, and included:

- ◦ Average counts per minute (CPM) per day
- ◦ Average vector magnitude per minute (VMPM) per day
- ◦ Average sedentary minutes/day (0-100 CPM) [45]
- ◦ Average light PA minutes/day (101–2019 CPM) [46]
- ◦ Average moderate-to-vigorous PA minutes/day (≥ 2,020 CPM) [46]

#### Park use variables

Park use was measured using three survey items adapted from a previously validated instrument [47, 48]. Participants reported park visit frequency in the past 30 days, and typical visit duration on weekdays and weekend days. Response options were converted from discrete categories to numeric values using a previously published protocol [49]. These inputs were used to construct three quasi-continuous variables: days visited and average minutes spent at the park on weekdays and weekend days. In addition, three dichotomous indicators were derived: visited the park ≥ once per week, spent ≥ 30 min at the park on a typical weekday, and spent ≥ 30 min at the park on a typical weekend day. All park use variables were drawn from post-renovation survey responses to assess and model the effects of naturally varying doses of exposure to the intervention through use of the renovated park spaces.

#### Sociodemographic variables

Sociodemographic information was collected at baseline and included the following: age, body mass index (BMI, in kg/m^2^), sex, race/ethnicity, annual household income, education, employment status, public housing, marital status, and children in household. Multivariate imputation by chained equations was used to impute a small number of missing baseline sociodemographic data (percent missing: age-0.9%, BMI-4.4%, race/ethnicity-2.0%, income-2.0%, education-2.9%). Given the low proportion of missingness (≤ 4.4% across variables) and the use of these variables as potential covariates, imputation was used to retain sample size and reduce potential bias due to missing sociodemographic data. Missing data points were filled with the most common predicted values across 50 imputed datasets using the full set of baseline sociodemographic variables as predictors. Convergence of the imputation algorithm was visually verified. A summary of imputed sociodemographic variables is available in *Additional File 1-Table S1*.

#### Weather variables

Daily weather time series data were obtained from the PRISM Data Explorer which provides high-resolution (4 km) daily weather statistics across the US [50]. Park site centroid coordinates were obtained using QGIS version 3.34.1 (https://qgis.org/). For each park site, weather data corresponding to the dates of accelerometer wear were retrieved from PRISM and merged with individual day-level accelerometry records. For each participant, daily weather metrics were aggregated into week-level summary measures over adherent days of accelerometer wear. These measures included the proportion of: rainy days (≥ 0.3 inches of precipitation), hot days (maximum temperature ≥ 85 °F), cold days (maximum temperature < 60 °F), and humid days (mean dew point > 55 °F) during the week of accelerometer wear.

### Statistical analysis

Descriptive statistics [mean (SD), n (%)] were calculated for sociodemographic variables, overall and stratified by intervention status. Chi-squared and t-tests assessed potential baseline differences in sociodemographic variables between groups. Descriptive statistics were also computed for PA outcomes and weather variables, stratified by intervention status and time (pre- and post-renovation).

To evaluate associations between the CPI park renovation intervention and changes in PA/SB over time, a difference-in-differences (DID) approach for repeated measures was implemented using linear mixed effects regression models. Models included fixed effects for intervention status (intervention vs. control), time (pre- vs. post-renovation), and their interaction (the DID estimator). Covariate adjustments included weather variables and baseline sociodemographic variables that were imbalanced between intervention and control groups. Weather variables were included in place of season or month indicators, as daily weather conditions during accelerometer wear are more proximally related to PA and can vary substantially within and across seasons.

To examine potential differences in the associations between park use patterns (i.e., duration and frequency) and changes in PA/SB, we extended the main models by including three-way interaction terms between intervention status, time, and park use variables. Post-hoc contrasts were used to obtain estimates for associations between park use and changes in PA/SB within intervention and control groups separately. This analysis was limited to participants who completed park use surveys.

All models accounted for within-participant and within-park correlations using random effects. Model-estimated changes in mean PA/SB outcomes within groups are reported with 95% confidence intervals (CIs). For DID estimators, 95% CIs and p-values are provided. Statistical significance was defined as α = 0.05 for primary analyses, and α = 0.1 for interaction models. Assumptions for linear regression were verified using residual plots. Analyses were conducted in R software version 4.5.1 (https://www.R-project.org) using *mice*, *lme4*, *lmerTest*, and *emmeans* packages [51–54].

### Sensitivity analysis

We examined two additional PA variables using alternative activity cutoffs: light PA minutes/day (101–759 CPM) and moderate-to-vigorous PA minutes/day (≥ 760 CPM) [45]. Multiple imputation was also used to impute park use variables for participants without follow-up surveys as an additional sensitivity analysis to assess potential bias due to missing park use data. Pooled results from multiply imputed datasets were compared with complete case analyses to evaluate the robustness of findings.

## Results

### Sample characteristics

344 adult participants enrolled in the PARCS study had ≥ 3 adherent days of accelerometer wear pre- and post-renovation, including 186 participants living near 31 parks that received renovations and 158 participants living near 21 control parks. The median number of participants per park site was 6 (IQR: 3–9).

The sample had a mean (SD) age of 42 (12) years and BMI of 30 (7) kg/m^2^ (Table 1). Most participants were female (81%), with 45% identifying as Latino/a, 38% as non-Latino/a Black, and 18% as other or multiracial (including White). Half of the sample reported an annual household income below $20,000; 45% had a high school education or less; and 55% were not employed. Additionally, 53% were NYCHA residents, 44% were never married, and 70% had one or more children living in the household. Intervention and control participants were similar across most baseline sociodemographic characteristics, with two exceptions: public housing status (47% of the intervention group were NYCHA residents vs. 59% of control group; χ^2^ *p* = 0.033) and presence of children in the household (65% of intervention group vs. 77% of control group; χ^2^ *p* = 0.010).

**Table 1:** Characteristics of adult PARCS study participants with complete accelerometry data (*n* = 344)

	Overall *n* = 344^*a*^	Intervention *n* = 186^*a*^	Control *n* = 158^*a*^	*p*-value^b^
Age	42 (12)	43 (12)	41 (12)	0.130
(Missing)	3	3	0	
Age Category				0.449
18-34y	103 (30%)	50 (27%)	53 (34%)	
35-49y	141 (41%)	78 (43%)	63 (40%)	
50-78y	97 (28%)	55 (30%)	42 (27%)	
(Missing)	3	3	0	
BMI	30 (7)	30 (7)	30 (7)	0.606
(Missing)	15	14	1	
BMI Category				0.113
Healthy/Underweight (BMI 16–25 kg/m^2^)	82 (25%)	35 (20%)	47 (30%)	
Overweight (BMI 25–29 kg/m^2^)	97 (29%)	56 (33%)	41 (26%)	
Obese (BMI 30–66 kg/m^2^)	150 (46%)	81 (47%)	69 (44%)	
(Missing)	15	14	1	
Sex				0.718
Female	278 (81%)	149 (80%)	129 (82%)	
Male	66 (19%)	37 (20%)	29 (18%)	
Race/Ethnicity				0.402
Latino/a	150 (45%)	81 (45%)	69 (44%)	
Non-Latino/a Black	128 (38%)	72 (40%)	56 (36%)	
Other or multiracial	59 (18%)	27 (15%)	32 (20%)	
(Missing)	7	6	1	
Annual Household Income				0.626
Less than $20,000	168 (50%)	88 (49%)	80 (51%)	
$20,000 or more	169 (50%)	93 (51%)	76 (49%)	
(Missing)	7	5	2	
Education				0.275
High school graduate or less	150 (45%)	84 (48%)	66 (42%)	
Some college or more	184 (55%)	92 (52%)	92 (58%)	
(Missing)	10	10	0	
Employment Status				0.887
Not employed	188 (55%)	101 (54%)	87 (55%)	
Employed or self-employed	156 (45%)	85 (46%)	71 (45%)	
Public Housing				0.033
Non-NYCHA resident	163 (47%)	98 (53%)	65 (41%)	
NYCHA resident	181 (53%)	88 (47%)	93 (59%)	
Marital Status				0.105
Never married	152 (44%)	87 (47%)	65 (41%)	
Married	113 (33%)	52 (28%)	61 (39%)	
Divorced, separated, or widowed	79 (23%)	47 (25%)	32 (20%)	
Children in Household				0.010
No children	102 (30%)	66 (35%)	36 (23%)	
One or more children	242 (70%)	120 (65%)	122 (77%)	

*Abbreviations:* *PARCS *Physical Activity and Redesigned Community Spaces, *BMI *Body Mass Index, *NYCHA *New York City Housing Authority

^a^Mean (SD); n (%)

^b^Welch Two Sample t-test; Pearson’s Chi-squared test

Weather conditions during accelerometer wear were significantly different pre- vs. post-renovation and by intervention status (*Additional File 1-Table S2*). In the overall sample, the post-renovation period was characterized by a greater proportion of rainy and cold days during accelerometer wear compared to the pre-renovation wave. Across both waves, participants in the intervention group wore accelerometers during a greater proportion of hot and humid days and a lower proportion of cold days relative to those in the control group.

### Associations between park renovations and changes in physical activity and sedentary behavior

**Fig. 1:**
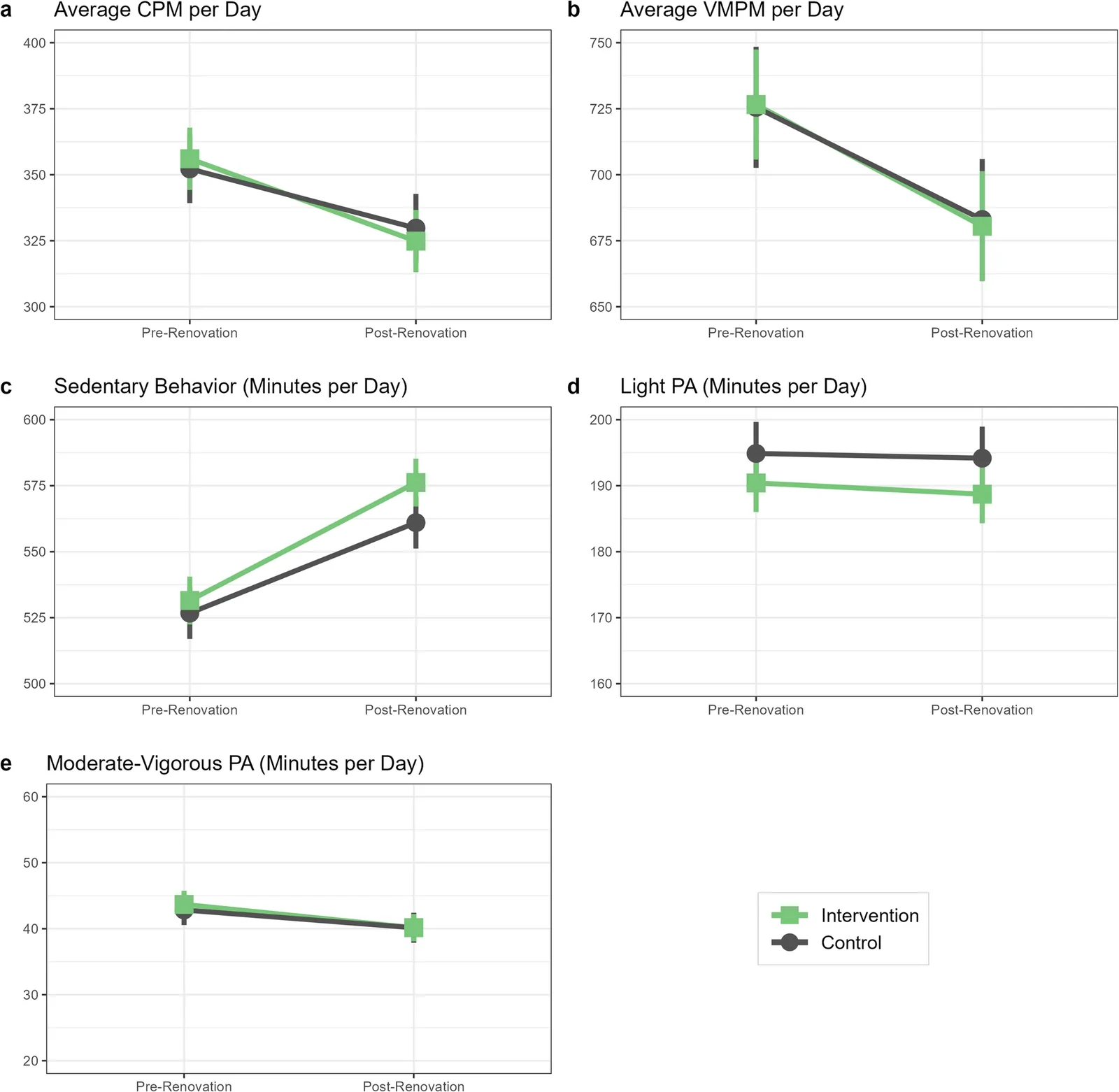
Mean daily physical activity and sedentary behavior levels (as measured by accelerometry) among adult PARCS study participants before and after the CPI park renovation intervention. Panel **a** = average CPM per day; panel **b** = average VMPM per day; panel **c** = sedentary behavior (in minutes per day); panel **d** = light PA (in minutes per day); panel **e** = moderate-vigorous PA (in minutes per day). Means by intervention group (green square = intervention, grey circle = control) and standard errors (vertical bars) were estimated using linear mixed effects regression models. Abbreviations: PARCS: Physical Activity and Redesigned Community Spaces; CPI: Community Parks Initiative; CPM: Counts per Minute; VMPM: Vector Magnitude per Minute; PA: Physical Activity

Across all outcomes, there were no differences in changes in PA/SB between participants living near parks receiving renovations compared to those living near control parks (Table 2; Fig. 1). Both intervention participants and control participants experienced substantial decreases in average CPM and VMPM in the post-renovation period compared to the pre-renovation period. Both groups also experienced an increase in sedentary minutes/day over time. There were no changes over time in either group in light or moderate-vigorous PA minutes/day.

**Table 2:** Changes in adult physical activity and sedentary behavior (as measured by accelerometry) over time among adult PARCS study participants in intervention (*n* = 186) vs. control (*n* = 158) groups

	Intervention (*n* = 186)			Control (*n* = 158)			Difference-in-Differences		
	Pre-Renovation^a^	Post-Renovation^a^	Change (Post-minus-Pre) (95% CI)	Pre-Renovation^a^	Post-Renovation^a^	Change (Post-minus-Pre) (95% CI)	Unadjusted DID Estimator (95% CI)^b^	Adjusted DID Estimator (95% CI)^b, c^	*p*-value for adjusted DID estimator^c^
CPM	355 (150)	324 (152)	-31.2 (-51.9, -10.4)	353 (123)	331 (128)	-22.5 (-45.0, 0.1)	-8.7 (-39.3, 21.9)	-7.8 (-39.6, 24.0)	0.630
VMPM	723 (252)	677 (269)	-46.1 (-81.3, -10.9)	727 (214)	685 (227)	-42.5 (-80.7, -4.3)	-3.6 (-55.6, 48.4)	0.2 (-53.7, 54.1)	0.994
Minutes/day of:									
Sedentary Behavior	532 (99)	576 (140)	44.7 (24.7, 64.6)	527 (98)	561 (144)	34.3 (12.6, 55.9)	10.4 (-19.0, 39.9)	7.5 (-23.0, 38.1)	0.627
Light PA	190 (57)	189 (63)	-1.7 (-9.9, 6.5)	195 (54)	194 (51)	-0.7 (-9.6, 8.1)	-1.0 (-13.0, 11.0)	-0.4 (-12.9, 12.0)	0.946
Moderate-Vigorous PA	44 (28)	40 (25)	-3.5 (-7.0, 0.0)	43 (21)	40 (21)	-2.7 (-6.5, 1.1)	-0.8 (-6.0, 4.3)	-0.6 (-6.0, 4.7)	0.813

*Abbreviations:* *PARCS *Physical Activity and Redesigned Community Spaces, *DID *Difference-in-Differences, *CPM *Counts per Minute, *VMPM *Vector Magnitude per Minute

^a^Mean (SD) (rounded to nearest integer)

^b^The DID estimator represents the difference in change in mean PA level measures in the intervention group minus the control group

^c^Estimated using linear mixed effects regression models adjusted for public housing, children in household, and weather variables

### Associations between park use patterns and changes in physical activity and sedentary behavior by intervention status

**Fig. 2:**
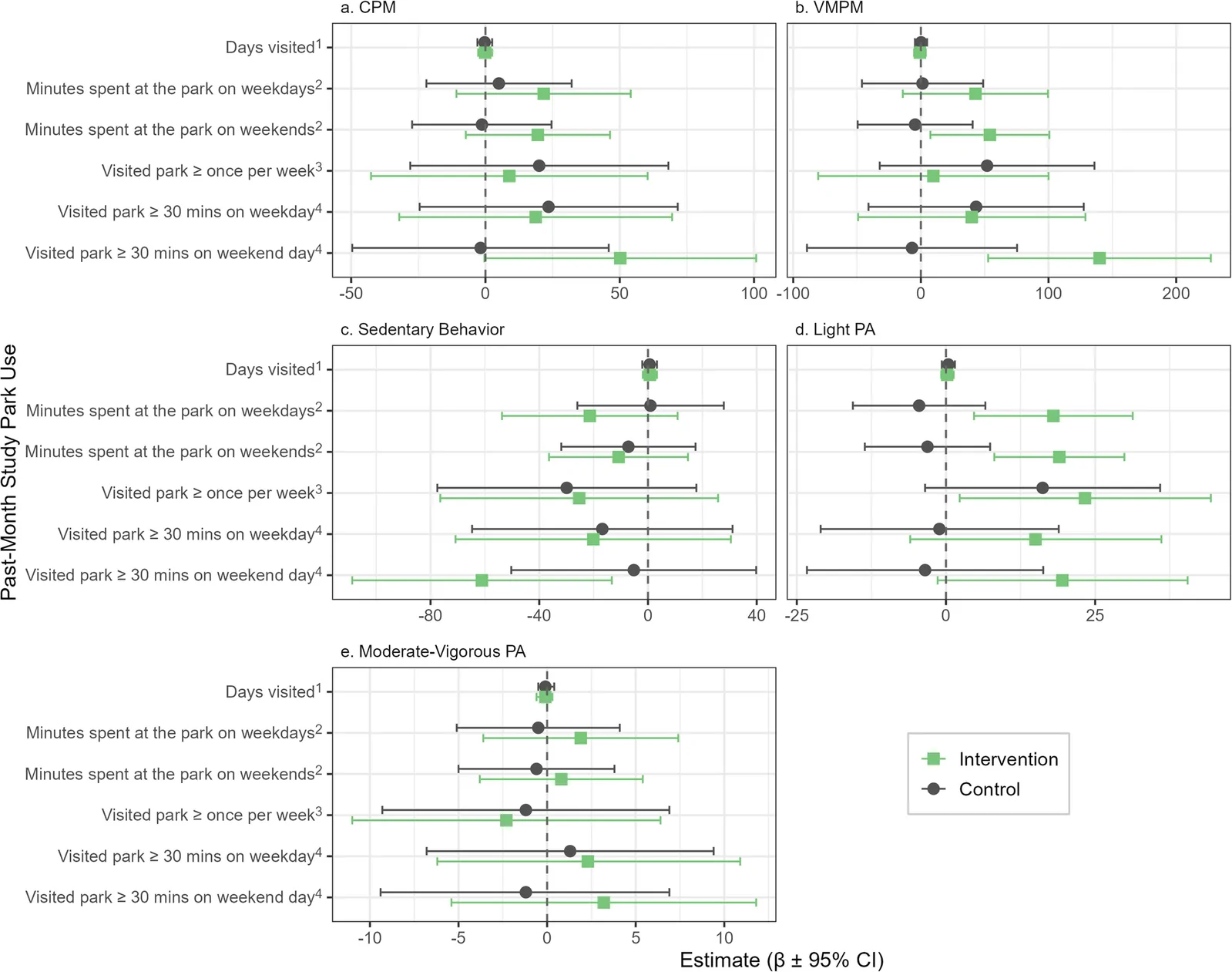
Associations between past-month study park use and changes in physical activity and sedentary behavior among adult PARCS study participants in intervention vs. control groups. Outcomes across panels include changes in: **a** = average CPM per day; **b** = average VMPM per day; **c** = sedentary behavior minutes per day; **d** = light PA minutes per day; **e** = moderate-vigorous PA minutes per day. Beta estimates (green square = intervention, grey circle = control) and 95% confidence intervals (horizontal bars) were estimated using linear mixed effects regression models. Footnotes: (1) Model estimates for days visited should be interpreted as change in PA level per additional park visit in the past month; (2) Model estimates for minutes spent at the park should be interpreted as change in PA level per 60-minute increase in usual time spent at the park on weekdays/weekend days; (3) Reference group = visited park < once per week; (4) Reference group = visited park < 30 min on weekday/weekend day. Abbreviations: PARCS: Physical Activity and Redesigned Community Spaces; CPM: Counts per Minute; VMPM: Vector Magnitude per Minute; PA: Physical Activity

Of the total sample, 240 participants (70%) completed post-renovation park use surveys and were included in the analysis examining associations between park use patterns and changes in PA/SB. Differences were observed between intervention and control groups in the associations between typical park use duration and changes over time in light PA minutes/day, average VMPM, and sedentary minutes/day (Table 3; Fig. 2). In the intervention group, each additional 60 min spent at the study park on weekdays was associated with 18.0 more minutes/day of light PA (95% CI 4.7, 31.3). On weekend days, 60 additional minutes of study park use in the intervention group corresponded to 19.0 more minutes/day of light PA (95% CI 8.1, 29.9) and a 54.1-unit increase in average VMPM (95% CI 7.5, 100.7). Furthermore, participants in the intervention group who reported visiting their study park for ≥ 30 min on a weekend day experienced a 140.0-unit greater increase in average VMPM (95% CI 52.7, 227.2) as well as a 61.1-minute (95% CI -108.8, -13.3) larger decrease in sedentary minutes/day compared to those with shorter visit durations (< 30 min). In the control group, there were no associations between park use duration on either weekdays or weekend days and changes in PA/SB.

**Table 3:** Associations between past-month study park use and changes in physical activity and sedentary behavior among adult PARCS study participants, stratified by intervention status (*n* = 186 intervention, *n* = 158 control)

	Days visited^a^	Minutes spent at the park on weekdays^b^	Minutes spent at the park on weekend days^b^	Visited park ≥ once per week^c^	Visited park ≥ 30 min on a weekday^d^	Visited park ≥ 30 min on a weekend day^d^
CPM						
Intervention	-0.1 (-2.7, 2.5)	21.7 (-10.8, 54.1)	19.5 (-7.3, 46.4)	8.9 (-42.6, 60.4)	18.7 (-32.1, 69.5)	50.2 (-0.4, 100.8)
Control	-0.3 (-3.0, 2.5)	5.0 (-22.0, 32.1)	-1.3 (-27.3, 24.6)	20.0 (-28.0, 68.1)	23.5 (-24.5, 71.6)	-1.8 (-49.6, 45.9)
p-value for Interaction	0.931	0.439	0.272	0.756	0.891	0.142
VMPM						
Intervention	-0.7 (-5.3, 3.9)	42.8 (-14.1, 99.6)	54.1 (7.5, 100.7)	9.8 (-80.4, 100.0)	39.9 (-49.1, 128.9)	140.0 (52.7, 227.2)
Control	0.2 (-4.6, 4.9)	1.3 (-46.1, 48.8)	-4.5 (-49.5, 40.6)	51.9 (-32.2, 136.0)	43.3 (-41.0, 127.6)	-6.9 (-89.2, 75.4)
p-value for Interaction	0.793	0.272	0.076	0.502	0.956	0.017
Sedentary Behavior						
Intervention	0.7 (-1.9, 3.3)	-21.4 (-53.7, 10.9)	-10.8 (-36.4, 14.7)	-25.3 (-76.4, 25.8)	-20.1 (-70.8, 30.5)	-61.1 (-108.8, -13.3)
Control	0.6 (-2.1, 3.3)	0.9 (-26.0, 27.9)	-7.2 (-31.9, 17.5)	-29.9 (-77.5, 17.8)	-16.8 (-64.7, 31.1)	-5.2 (-50.3, 39.8)
p-value for Interaction	0.948	0.297	0.840	0.898	0.924	0.096
Light PA						
Intervention	0.2 (-0.8, 1.3)	18.0 (4.7, 31.3)	19.0 (8.1, 29.9)	23.3 (2.3, 44.4)	15.0 (-6.0, 36.1)	19.5 (-1.4, 40.5)
Control	0.4 (-0.7, 1.5)	-4.5 (-15.6, 6.6)	-3.1 (-13.6, 7.4)	16.2 (-3.5, 35.9)	-1.1 (-21.0, 18.9)	-3.5 (-23.3, 16.3)
p-value for Interaction	0.842	0.011	0.004	0.626	0.276	0.116
Moderate-Vigorous PA						
Intervention	-0.1 (-0.6, 0.3)	1.9 (-3.6, 7.4)	0.8 (-3.8, 5.4)	-2.3 (-11.0, 6.4)	2.3 (-6.2, 10.9)	3.2 (-5.4, 11.8)
Control	-0.1 (-0.5, 0.4)	-0.5 (-5.1, 4.1)	-0.6 (-5.0, 3.8)	-1.2 (-9.3, 6.9)	1.3 (-6.8, 9.4)	-1.2 (-9.4, 6.9)
p-value for Interaction	0.885	0.513	0.663	0.854	0.868	0.464

*Abbreviations:* *CPM *Counts per Minute, *VMPM *Vector Magnitude per Minute, *PA *Physical Activity

^a^Model estimates for days visited should be interpreted as change in PA level per additional park visit in the past month

^b^Model estimates for minutes spent at the park should be interpreted as change in PA level per 60-minute increase in usual time spent at the park on weekdays/weekend days

^c^Reference group = visited park < once per week

^d^Reference group = visited park < 30 min on weekday/weekend day

### Sensitivity analysis

Model estimates derived using alternative cutoffs to define light and moderate-to-vigorous PA produced results consistent with the primary analysis and did not alter the overall conclusions (*Additional File 1-Tables S3 & S4*). Additionally, associations between past-month study park use and adult PA/SB by intervention status remained robust in the multiply imputed sample (*n* = 302), showing no meaningful differences from the complete case analysis (*n* = 240) (*Additional File 1-Table S5*).

## Discussion

In one of the largest park quality improvement evaluations to-date, we measured associations between citywide park renovations and changes in accelerometry-based PA and SB among 344 adults living near 31 renovated parks and 21 sociodemographically-matched control parks in low-income NYC neighborhoods. Overall, we found no association between park renovations and changes in daily PA/SB at the individual level in a sample that was predominantly low-income, Latino/a and Black. However, longer typical park visit duration was associated with significant increases in total PA volume and light PA, and with reduced sedentary time among residents near renovated parks but not control parks. These findings suggest that while park renovation alone may not directly increase PA or reduce SB, high-quality, renovated park spaces may be a necessary pre-condition for the effect of higher park use on increased PA and reduced SB among adults in disadvantaged urban communities.

Few intervention studies have examined individual-level changes in PA or used accelerometry-based measures to evaluate the effects of park improvements on adult PA. To our knowledge, our study is the first to exclusively evaluate park renovations using accelerometry-based measures of PA/SB, and is one of few studies evaluating renovations at more than ten sites. At the time of publishing, we have identified only one prior study that has evaluated individual-level longitudinal changes in PA/SB attributable to park improvements using accelerometry-based measures, which found no changes in PA/SB attributable to an intervention comprising park and playground improvements paired with a cable car installation in Bogotá, Colombia [25].

A recent systematic review identified 15 park renovation intervention studies published from 2009 to 2024 that evaluated at least one PA outcome [23]. Among 10 studies evaluating outcomes at the park-level, eight showed at least one significant positive effect of park improvements on the PA levels of park users [25, 33, 55–60], while two showed no significant changes in PA attributable to park improvements [61, 62]. In contrast, the same review identified only three studies that assessed individual-level PA outcomes, all of which showed either null or mixed findings [24–26]. One possible explanation for the discrepancy in findings between park-level and individual-level studies is that these study designs capture fundamentally different populations and outcomes. Park-level studies assess the activity of individuals already present in the park, and therefore are more likely to detect increases in park-based activity following improvements. In contrast, individual-level studies, including the present study, evaluate changes in overall PA among residents of the surrounding neighborhood, which may not increase even if activity within parks improves. Consistent with this interpretation is the hypothesis that park and trail improvements may increase activity within parks without increasing total PA due to displacement of activity from other settings (e.g., homes, gyms, or other park sites) [23, 63, 64]. More broadly, differences across studies highlight the challenge of comparing findings across the literature, as park renovation interventions vary substantially in study design and implementation context [23], including variation in the type and scope of park improvements, neighborhood and population characteristics, levels and sustainment of investment, and features of the surrounding built and social environment.

While CPI renovations alone were not associated with increased PA overall, residents living near renovated parks who reported longer typical durations of visits to their study park experienced larger improvements in PA/SB over time, whereas visit duration was unrelated to changes in PA/SB among residents living near control parks. Similarly, in a previous study, we showed that residents living near renovated CPI parks who used them frequently (≥ once/week) had a significantly larger decrease in perceived stress over time compared to those with infrequent renovated park use (< once/week), while park use was unrelated to changes in perceived stress among residents living near control parks [41]. The differential effects we observed between intervention and control park neighborhoods—where the same level of park use yielded beneficial associations with PA/SB and perceived stress at renovated parks, but not in control parks—demonstrates that quality may be key to unlocking the potential public health benefits of park use. This idea is further supported by emerging evidence in the literature where beneficial associations between park use and health outcomes have been observed in the context of high-quality parks [11, 30, 65], whereas past studies that did not control for park quality have shown mixed associations [9, 66], including evidence suggesting that low-quality parks in disrepair or with incivilities may be associated with worse health and social outcomes [16, 17]. In the case of PA, a plausible explanation for this phenomenon is that high-quality renovated parks such as the CPI parks offer a greater diversity of PA-supporting features that may enable and sustain activity during longer park visits, as several prior studies have shown that both the number and type of park features, particularly facilities such as walking trails and activity spaces, are associated with higher park-based PA levels [15, 67, 68]. Spending additional time at parks without diverse PA-supporting amenities or with significant barriers to their use (e.g., safety concerns or poor conditions) may have less effect on overall PA levels [19, 68].

Other previous research evaluating the CPI in NYC showed that park renovations were associated with increased park usership [33], the amount of time neighborhood residents typically spent at parks [34], as well as satisfaction with the renovated park spaces [34]. The lack of direct effect of park renovations observed in the present study suggest that park renovations alone may not be sufficient to generate meaningful changes in PA/SB among adults. Instead, the health benefits associated with renovated park spaces appear to depend on the degree to which residents engage with these environments, as well as the populations that are reached. Prior work from our team has demonstrated that the benefits of CPI on park use was not uniform across all groups, with the largest improvements in park use seen among adults with children and Latin/o adults [34]. Our findings collectively point to the need to move beyond evaluating the presence of improved built environments alone to considering how they are used over time as a key dimension of exposure to the intervention. As such, efforts to maximize the public health impact of park renovation interventions may require complementary strategies such as tailored programming, co-design, and other community-engaged activation efforts to support more frequent and sustained engagement with renovated park spaces across diverse groups [11, 34].

Furthermore, the observed decreases in total PA volume and increases in sedentary time observed in both intervention and control groups may reflect broader contextual influences beyond the control of the study. Maturational declines in PA across adulthood are well documented and could partly explain the observed downward trends over time [7, 69, 70]. In addition, differences in weather conditions between pre- and post-renovation periods could have also influenced PA levels independent of park renovations. Although adjustment for weather in the main DID models was a key analytical strength of this study (reducing the effects of confounding due to varying weather conditions across time and treatment groups), weather-related factors may still have contributed to the overall trends in PA/SB across groups and should be considered when interpreting the findings.

This study has several limitations. We relied on self-reported measures of park use, which may be subject to recall bias and misreporting. While accelerometry provided objective measures of overall PA/SB, it did not allow us to distinguish park-based activity from activity occurring in other settings. As a result, we were unable to directly link PA levels to time spent in the park. Future studies incorporating geolocation data from GPS-enabled smartphones or other wearable devices could allow for more precise linkage between park use and PA [71]. In addition, Participants were recruited as a convenience sample and may not be fully representative of the underlying populations in the study catchment areas, which may limit generalizability [41]. However, because the sampling approach was applied consistently across intervention and control groups, comparisons of changes in PA outcomes between the two groups remain valid. Furthermore, the study’s modest sample size likely reduced our power to detect some associations our analyses; however the sample was sizeable for typical longitudinal intervention studies employing accelerometry for PA/SB data collection [72, 73]. Finally, only a single pre-renovation round of data was collected, so the parallel trends assumption for causal inference using DID could not be empirically verified, although previous analysis of neighborhood census-block sociodemographic trends prior to CPI showed no differential changes in population characteristics between intervention and control sites [34, 41].

## Conclusions

Park renovations alone were not associated with changes in accelerometer-measured adult PA/SB, but among residents living near renovated parks, longer typical visit durations were associated with increased total PA volume and light PA, and reduced sedentary time. These findings suggest that high-quality park environments may be a necessary precondition for the PA/SB benefits of higher park use, and that renovations should be paired with efforts to promote longer park visits to fully realize their health benefits.

## Supplementary Information

Supplementary Material 1.

## Data Availability

The datasets used and/or analyzed during the current study are available from the corresponding author on reasonable request.

## Abbreviations

BMI: Body mass index
CI: Confidence interval
CPI: Community Parks Initiative
CPM: Counts per minute
DID: Difference-in-differences
IQR: Interquartile range
NYC: New York City
NYCHA: New York City Housing Authority
PA: Physical activity
PARCS: Physical Activity and Redesigned Community Spaces
SB: Sedentary behavior
SD: Standard deviation
US: United States
VMPM: Vector magnitude per minute
