# Supplementary material for: Park renovations in low-income New York City neighborhoods and changes in adult physical activity and sedentary behavior: a quasi-experimental study
Source: Int J Behav Nutr Phys Act. 2026 Aug 3;23:103. doi: 10.1186/s12966-026-01958-1 (PMC13726213; doi:10.1186/s12966-026-01958-1)
Supplement: Supplementary file 1 [file 12966_2026_1958_MOESM1_ESM.docx]

**Park renovations in low-income New York City neighborhoods and changes in adult physical activity and sedentary behavior: A quasi-experimental study**

Additional File 1

Authors: Rachel L. Thompson, Kelly R. Evenson, Katarzyna E. Wyka, Lorna E. Thorpe, Glen D. Johnson, Brian T. Pavilonis & Terry T.-K. Huang

**Table of Contents**

**Table S1.** Imputed sociodemographic characteristics of adult PARCS study participants with complete accelerometry data (n = 344)

**Table S2.** Summary of weather conditions during accelerometer wear pre- and post-renovation by intervention status (n = 186 intervention, n = 158 control)

**Table S3.** Changes in adult physical activity and sedentary behavior (as measured by accelerometry) over time among adult PARCS study participants in intervention (n = 186) vs. control (n = 158) groups, using alternative physical activity level cutoffs published by Matthews et al.

**Table S4.** Associations between past-month study park use and changes in adult physical activity, stratified by intervention status (n = 186 intervention, n = 158 control), using alternative physical activity level cutoffs published by Matthews et al.

**Table S5.** Associations between past-month study park use and changes in adult physical activity, stratified by intervention status, imputed results (n = 302).

**Table S1.** Imputed sociodemographic characteristics of adult PARCS study participants with complete accelerometry data (n = 344)

|  | **Overall**  n = 344*^1^* | **Intervention**  n = 186*^1^* | **Control**  n = 158*^1^* | **p-value***^2^* |
| --- | --- | --- | --- | --- |
| **Age** | 42 (12) | 43 (12) | 41 (12) | 0.114 |
| **Age Category** |  |  |  | 0.398 |
| 18-34y | 103 (30%) | 50 (27%) | 53 (34%) |  |
| 35-49y | 143 (42%) | 80 (43%) | 63 (40%) |  |
| 50-78y | 98 (28%) | 56 (30%) | 42 (27%) |  |
| **BMI** | 30 (7) | 30 (7) | 30 (7) | 0.679 |
| **BMI Category** |  |  |  | 0.107 |
| Healthy/Underweight (BMI 16-25 kg/m^2^) | 87 (25%) | 39 (21%) | 48 (30%) |  |
| Overweight (BMI 25-29 kg/m^2^) | 102 (30%) | 61 (33%) | 41 (26%) |  |
| Obese (BMI 30-66 kg/m^2^) | 155 (45%) | 86 (46%) | 69 (44%) |  |
| **Sex** |  |  |  | 0.718 |
| Female | 278 (81%) | 149 (80%) | 129 (82%) |  |
| Male | 66 (19%) | 37 (20%) | 29 (18%) |  |
| **Race/Ethnicity** |  |  |  | 0.353 |
| Latino | 153 (44%) | 84 (45%) | 69 (44%) |  |
| Non-Latino Black | 132 (38%) | 75 (40%) | 57 (36%) |  |
| Other or multiracial | 59 (17%) | 27 (15%) | 32 (20%) |  |
| **Annual Household Income** |  |  |  | 0.582 |
| Less than $20,000 | 173 (50%) | 91 (49%) | 82 (52%) |  |
| $20,000 or more | 171 (50%) | 95 (51%) | 76 (48%) |  |
| **Education** |  |  |  | 0.259 |
| High school graduate or less | 155 (45%) | 89 (48%) | 66 (42%) |  |
| Some college or more | 189 (55%) | 97 (52%) | 92 (58%) |  |
| **Employment Status** |  |  |  | 0.887 |
| Not employed | 188 (55%) | 101 (54%) | 87 (55%) |  |
| Employed or self-employed | 156 (45%) | 85 (46%) | 71 (45%) |  |
| **Public Housing** |  |  |  | 0.033 |
| Non-NYCHA resident | 163 (47%) | 98 (53%) | 65 (41%) |  |
| NYCHA resident | 181 (53%) | 88 (47%) | 93 (59%) |  |
| **Marital Status** |  |  |  | 0.105 |
| Never married | 152 (44%) | 87 (47%) | 65 (41%) |  |
| Married | 113 (33%) | 52 (28%) | 61 (39%) |  |
| Divorced, separated, or widowed | 79 (23%) | 47 (25%) | 32 (20%) |  |
| **Children in Household** |  |  |  | 0.010 |
| No children | 102 (30%) | 66 (35%) | 36 (23%) |  |
| One or more children | 242 (70%) | 120 (65%) | 122 (77%) |  |
| *^1^*Mean (SD); n (%) | | | | |
| *^2^*Welch Two Sample t-test; Pearson's Chi-squared test  Abbreviations – PARCS: Physical Activity and Redesigned Community Spaces; BMI: Body Mass Index;  NYCHA: New York City Housing Authority | | | | |

**Table S2.** Summary of weather conditions during accelerometer wear pre- and post-renovation by intervention status (n = 186 intervention, n = 158 control)

|  | **Overall Sample** | | | **Pre-Renovation by Group** | | | **Post-Renovation by Group** | | |
| --- | --- | --- | --- | --- | --- | --- | --- | --- | --- |
| **Proportion of days when accelerometer was worn that were:** | **Pre-Renovation**  n = 344*^1^* | **Post-Renovation**  n = 344*^1^* | **p-value***^2^* | **Intervention**  n = 186*^1^* | **Control**  n = 158*^1^* | **p-value***^2^* | **Intervention**  n = 186*^1^* | **Control**  n = 158*^1^* | **p-value***^2^* |
| Rainy (≥ 0.3 inches precipitation) | 0.15 (0.15) | 0.20 (0.18) | <0.001 | 0.14 (0.15) | 0.16 (0.15) | 0.374 | 0.21 (0.20) | 0.19 (0.16) | 0.279 |
| Hot (max temp ≥ 85°F) | 0.20 (0.29) | 0.18 (0.28) | 0.353 | 0.33 (0.32) | 0.05 (0.12) | <0.001 | 0.22 (0.29) | 0.13 (0.25) | 0.004 |
| Cold (max temp < 60°F) | 0.22 (0.35) | 0.36 (0.43) | <0.001 | 0.08 (0.26) | 0.38 (0.38) | <0.001 | 0.24 (0.40) | 0.50 (0.43) | <0.001 |
| Humid (mean dew point temp > 55°F) | 0.55 (0.42) | 0.49 (0.44) | 0.078 | 0.75 (0.32) | 0.30 (0.38) | <0.001 | 0.61 (0.42) | 0.34 (0.41) | <0.001 |
| *^1^*Mean (SD) | | | | | | | | | |
| *^2^*Welch Two Sample t-test |  |  |  |  |  |  |  |  |  |

**Table S3.** Changes in adult physical activity and sedentary behavior (as measured by accelerometry) over time among adult PARCS study participants in intervention (n = 186) vs. control (n = 158) groups, using alternative physical activity level cutoffs published by Matthews et al.

|  | **Intervention**  **(n = 186)** | | | **Control**  **(n = 158)** | | | **Difference-in-Differences** | | |
| --- | --- | --- | --- | --- | --- | --- | --- | --- | --- |
|  | **Pre-Renovation**^1^ | **Post-Renovation**^1^ | **Change**  **(Pre-minus-Post)**  **(95% CI)** | **Pre-Renovation**^1^ | **Post-Renovation**^1^ | **Change**  **(Pre-minus-Post)**  **(95% CI)** | **Unadjusted DID Estimator (95% CI)^2^** | **Adjusted DID Estimator (95% CI)^2,3^** | **p-value for adjusted DID estimator^3^** |
| **Minutes/day of:** |  |  |  |  |  |  |  |  |  |
| Light PA | 124 (36) | 125 (40) | 0.8 (-4.4, 6.0) | 131 (36) | 131 (34) | -0.3 (-5.9, 5.3) | 1.1 (-6.6, 8.8) | 0.8 (-7.2, 8.7) | 0.848 |
| Moderate-Vigorous PA | 110 (44) | 104 (46) | -6.0 (-12.1, 0.0) | 107 (36) | 104 (37) | -3.1 (-9.7, 3.5) | -2.9 (-11.9, 6.1) | -2.0 (-11.3, 7.3) | 0.676 |
| ^1^Mean (SD) (rounded to nearest integer)  ^2^The DID estimator represents the difference in change in mean PA level measures in the intervention group minus the control group  ^2^Estimated using linear mixed effects regression models adjusted for public housing, children in household, and weather variables  Abbreviations – PARCS: Physical Activity and Redesigned Community Spaces; DID: Difference-in-Differences; PA: Physical Activity | | | | | | | | | |

**Table S4.** Associations between past-month study park use and changes in physical activity and sedentary behavior among adult PARCS study participants, stratified by intervention status (n = 186 intervention, n = 158 control), using alternative physical activity level cutoffs published by Matthews et al.

|  | **Days visited^1^** | **Minutes spent at the park on weekdays^2^** | **Minutes spent at the park on weekend days^2^** | **Visited park ≥ once per week^3^** | **Visited park ≥ 30 mins on a weekday^4^** | **Visited park ≥ 30 mins on a weekend day^4^** |
| --- | --- | --- | --- | --- | --- | --- |
| **Light PA** |  |  |  |  |  |  |
| Intervention | 0.0 (-0.7, 0.7) | 9.5 (0.9, 18.2) | 11.4 (4.4, 18.5) | 12.0 (-1.6, 25.7) | 4.0 (-9.7, 17.6) | 7.7 (-5.8, 21.3) |
| Control | 0.2 (-0.5, 1.0) | -3.8 (-11.0, 3.4) | -1.7 (-8.5, 5.1) | 10.7 (-2.1, 23.4) | -2.4 (-15.3, 10.5) | -2.8 (-15.6, 9.9) |
| p-value for Interaction | 0.601 | 0.020 | 0.009 | 0.888 | 0.505 | 0.264 |
| **Moderate-Vigorous PA** |  |  |  |  |  |  |
| Intervention | 0.2 (-0.6, 0.9) | 10.4 (1.0, 19.8) | 8.4 (0.6, 16.2) | 9.0 (-5.9, 24.0) | 13.4 (-1.4, 28.1) | 15.0 (0.2, 29.7) |
| Control | 0.1 (-0.7, 0.9) | -1.2 (-9.0, 6.7) | -1.9 (-9.5, 5.6) | 4.3 (-9.6, 18.3) | 2.7 (-11.3, 16.6) | -1.9 (-15.8, 12.0) |
| p-value for Interaction | 0.909 | 0.063 | 0.061 | 0.652 | 0.300 | 0.103 |
| ^1^Model estimates for days visited should be interpreted as change in PA level per additional park visit in the past month  ^2^Model estimates for minutes spent at the park should be interpreted as change in PA level per 60-minute increase in usual time spent at the park on weekdays/weekend days  ^3^Reference group = visited park < once per week  ^4^Reference group = visited park < 30 minutes on weekday/weekend day  Abbreviations – PA: Physical Activity | | | | | | |

**Table S5.** Associations between past-month study park use and changes in physical activity and sedentary behavior among adult PARCS study participants, stratified by intervention status, imputed results (n = 302). Multiple imputation by chained equations was used to impute missing park use variables at follow-up. The sample was subset to include only those with complete park use at follow-up (n = 240) or those with missing park-use at follow-up but non-missing park use at baseline (n = 62). Twenty-five imputed datasets were generated from the original data using the full set of sociodemographic and baseline park use variables as predictors. Model results below were pooled across the 25 imputed datasets.

|  | **Days visited^1^** | **Minutes spent at the park on weekdays^2^** | **Minutes spent at the park on weekend days^2^** | **Visited park ≥ once per week^3^** | **Visited park ≥ 30 mins on a weekday^4^** | **Visited park ≥ 30 mins on a weekend day^4^** |
| --- | --- | --- | --- | --- | --- | --- |
| **CPM** |  |  |  |  |  |  |
| Intervention | 0.0 (-2.4, 2.4) | 16.4 (-15.1, 47.8) | 15.9 (-10.4, 42.2) | 7.8 (-41.6, 57.1) | 13.1 (-34.5, 60.5) | 35.2 (-14.8, 85.2) |
| Control | -0.3 (-2.9, 2.4) | 6.9 (-20.3, 34.1) | -4.1 (-29.7, 21.5) | 17.3 (-30.7, 65.2) | 23.1 (-24.2, 70.4) | -5.6 (-52.3, 41.1) |
| p-value for Interaction | 0.876 | 0.658 | 0.289 | 0.783 | 0.769 | 0.243 |
| **VMPM** |  |  |  |  |  |  |
| Intervention | -0.9 (-5.2, 3.5) | 31.4 (-23.0, 85.7) | 42.5 (-2.6, 87.6) | 1.2 (-85.5, 87.9) | 27.3 (-55.0, 109.6) | 99.5 (15.1, 184.0) |
| Control | 0.1 (-4.5, 4.6) | 7.6 (-39.4, 54.5) | -8.3 (-52.5, 35.8) | 45.0 (-37.7, 127.7) | 45.3 (-36.1, 126.8) | -11.2 (-91.5, 69.1) |
| p-value for Interaction | 0.773 | 0.517 | 0.118 | 0.466 | 0.761 | 0.063 |
| **Sedentary Behavior (Matthews)** |  |  |  |  |  |  |
| Intervention | 0.7 (-1.8, 3.2) | -18.7 (-51.4, 14.0) | -9.0 (-34.7, 16.7) | -14.3 (-64.4, 35.9) | -18.5 (-66.7, 29.7) | -45.8 (-93.0, 1.4) |
| Control | 1.2 (-1.5, 4.0) | -7.1 (-34.1, 20.0) | -3.4 (-29.0, 22.3) | -14.0 (-61.9, 34.0) | -19.7 (-67.6, 28.3) | -1.1 (-47.6, 45.3) |
| p-value for Interaction | 0.768 | 0.594 | 0.763 | 0.993 | 0.973 | 0.191 |
| **Light PA (Troiano)** |  |  |  |  |  |  |
| Intervention | 0.0 (-0.9, 1.0) | 14.9 (2.4, 27.4) | 16.3 (5.7, 26.9) | 15.5 (-4.8, 35.9) | 12.6 (-7.3, 32.6) | 14.8 (-5.2, 34.7) |
| Control | 0.4 (-0.6, 1.5) | -2.4 (-13.3, 8.4) | -3.0 (-13.1, 7.2) | 15.2 (-4.0, 34.3) | 1.1 (-18.1, 20.2) | -2.8 (-21.9, 16.2) |
| p-value for Interaction | 0.621 | 0.038 | 0.010 | 0.978 | 0.408 | 0.210 |
| **Moderate-Vigorous PA (Troiano)** |  |  |  |  |  |  |
| Intervention | 0.0 (-0.4, 0.4) | 1.0 (-4.1, 6.1) | 0.3 (-4.1, 4.6) | -1.0 (-9.1, 7.1) | 1.0 (-6.8, 8.8) | 1.2 (-7.1, 9.6) |
| Control | 0.0 (-0.5, 0.4) | 0.1 (-4.4, 4.6) | -0.6 (-4.8, 3.6) | -0.9 (-8.8, 7.1) | 2.2 (-5.6, 10.1) | -1.2 (-8.9, 6.6) |
| p-value for Interaction | 0.977 | 0.807 | 0.776 | 0.983 | 0.831 | 0.678 |
| **Light PA (Matthews)** |  |  |  |  |  |  |
| Intervention | -0.1 (-0.7, 0.6) | 7.8 (-0.6, 16.2) | 9.6 (2.8, 16.5) | 7.9 (-5.2, 21.1) | 3.8 (-9.6, 17.2) | 5.7 (-7.4, 18.8) |
| Control | 0.2 (-0.5, 0.9) | -2.1 (-9.1, 5.0) | -1.7 (-8.2, 4.9) | 9.7 (-2.8, 22.2) | -0.5 (-12.9, 11.9) | -2.3 (-14.6, 10.0) |
| p-value for Interaction | 0.515 | 0.076 | 0.020 | 0.844 | 0.644 | 0.381 |
| **Moderate-Vigorous PA (Matthews)** |  |  |  |  |  |  |
| Intervention | 0.1 (-0.6, 0.8) | 8.0 (-0.9, 16.9) | 6.9 (-0.8, 14.6) | 6.6 (-7.9, 21.1) | 9.8 (-3.9, 23.6) | 10.3 (-4.2, 24.7) |
| Control | 0.2 (-0.6, 0.9) | -0.3 (-8.1, 7.5) | -1.9 (-9.2, 5.4) | 4.5 (-9.1, 18.2) | 3.7 (-9.9, 17.6) | -1.7 (-15.2, 11.8) |
| p-value for Interaction | 0.936 | 0.170 | 0.100 | 0.838 | 0.535 | 0.231 |
| ^1^Model estimates for days visited should be interpreted as change in PA level per additional park visit in the past month  ^2^Model estimates for minutes spent at the park should be interpreted as change in PA level per 60-minute increase in usual time spent at the park on weekdays/weekend days  ^3^Reference group = visited park < once per week  ^4^Reference group = visited park < 30 minutes on weekday/weekend day; Abbreviations – CPM: Counts per Minute; VMPM: Vector Magnitude per Minute; PA: Physical Activity | | | | | | |
